# Living with a rare disorder: a systematic review of the qualitative literature

**DOI:** 10.1002/mgg3.315

**Published:** 2017-07-23

**Authors:** Charlotte von der Lippe, Plata S. Diesen, Kristin B. Feragen

**Affiliations:** ^1^ Centre for Rare Disorders Oslo University Hospital, Rikshospitalet P.B. 4950 Nydalen Oslo 0424 Norway

**Keywords:** adult, patient experiences, psychosocial, qualitative research, rare diseases, systematic review

## Abstract

**Background:**

Individuals with rare diseases may face challenges that are different from those experienced in more common medical conditions. A wide range of different rare conditions has resulted in a myriad of studies investigating the specificities of the diagnosis in focus. The shared psychological experiences of individuals with a rare condition, however, have not been reviewed systematically.

**Methods:**

We performed a systematic review, including qualitative studies on adults, published between 2000 and 2016. Papers including more than one rare genetic or nongenetic diagnosis were included. Studies based on single diagnoses were excluded except for four specific conditions: hemophilia (bleeding disorder), phenylketonuria (metabolic disorder), Fabry disease (lysosomal storage disorder), and epidermolysis bullosa (skin disorder).

**Results:**

The review identified 21 studies. Findings were synthesized and categorized according to three main themes: (1) Consequences of living with a rare disorder, (2) Social aspects of living with a rare disorder, and (3) Experiences with the health care system. Findings point to several unique challenges, such as the psychological, medical, and social consequences of a lack of knowledge about the condition in health care and social settings.

**Conclusion:**

The findings highlight the need for more research on the shared psychological and social impact of living with a rare diagnosis across conditions, in order to identify risk factors and inform clinical practice.

## Introduction

Rare diseases, also known as orphan diseases, are medical conditions that affect only a very limited number of individuals. No single definition of rare disease prevalence exists, and the criterions range from 1:2000 in the European Union (“http://www.eurordis.org/content/what-rare-disease,” 09/09/2014), to 1:10 000 in Norway (“https://helsenorge.no/sjeldne-diagnoser/hva-er-en-sjelden-diagnose,” 2016). In the USA, a disease is considered rare if it affects <200,000 (~1:1600) affected individuals (“https://rarediseases.org/,” 2016). Most rare diseases are genetic (Boycott et al. 2013), often chronic, and may imply a high level of both physical and psychological suffering (for a review, see (Cohen and Biesecker 2010; Waldboth et al. 2016))***.*** Depending on the specificity of the condition, more or less efficient treatments exist, but common to almost all, is that there is no cure for the disease (Institute of Medicine Committee on Accelerating Rare Diseases 2010). There are between 6000 and 8000 rare diseases (“http://www.eurordis.org/content/what-rare-disease,” 09/09/2014), which means that although each disease is rare, it is not rare to have a rare disease. It has been estimated that rare conditions may affect as many as 30 million Europeans and 25 million North Americans (Haffner et al. 2002; Dodge et al. 2011). Hence, in spite of the low prevalence of rare conditions, many people across the world have to live and cope with the medical, psychological, and social consequences of their condition. Due to a low prevalence, knowledge about rare diseases is sparse both in society and among healthcare professionals. One suggested way in improving the situation of people with rare conditions is to increase the awareness of rare diseases throughout society (Wastfelt et al. 2006; Dodge et al. 2011), and more specifically within the healthcare system.

Given the enormous range of studies based on single diagnoses, knowledge about shared experiences across rare conditions is hampered. Review papers investigating people with rare disorders’ psychological and social experiences across conditions could be one way of addressing the challenges of lack of knowledge in the health care system, by summarizing, discussing, and presenting shared experiences and their consequences in everyday life.

Clinical experience and personal accounts from people living with rare diseases (Kole and Faurisson 2009) provide many examples of a lack of knowledge in society and in the health care system. Consequences may be diagnostic mistakes, delays in diagnosis, and lack of information of high quality (Kole and Faurisson 2009; Nutt and Limb 2011; Molster et al. 2016). However, few if any studies have addressed this question systematically, and a synthesis of findings across rare conditions and studies have to the authors’ knowledge not been performed.

A systematic review of the quantitative literature on quality of life in individuals with rare genetic conditions (Cohen and Biesecker 2010), revealed that a large number of quality of life studies focus on disease‐related variables, keeping a bio‐medical model that fails to incorporate the patient‐centered perspective and hence explore the psychosocial impact of a rare medical condition. However, some studies were identified (Cohen and Biesecker 2010), all revealing strong correlations between psychosocial factors and quality of life that should be investigated further in future research. Qualitative methodology, a method for gaining deeper insight into people's experiences and seeking to understand the meaning or nature of the experiences (Strauss and Corbin 1990) is ideally suited for investigating the psychological, emotional, and social specifics of living with a rare disorder. Nonetheless, qualitative studies are less common than quantitative methods in health research more generally, possibly because these approaches are not well understood and/or because lesser value is placed on them (Nelson 2009). Hence, there is a lack of qualitative research exploring the patients’ experiences of living with a rare disease, exploring whether individuals with rare diseases face challenges that are qualitatively different from those experienced by people with more common medical conditions. Further, there is a lack of studies including several diagnoses, and hence exploring similarities and differences across conditions in a psychological perspective. Last, there is a lack of literature reviews summarizing experiences of individuals living with a rare diagnosis across conditions. In summary, the uniqueness of the psychological experiences of individuals with a rare condition has not been reviewed systematically.

### Aims

In order to address the lack of generalized and synthesized knowledge regarding people's experiences with living with a rare disease, a systematic review of the available qualitative research was conducted. The aim was to address shared experiences across rare diagnoses, without any focus on specific challenges for single diagnoses.

The aim of the systematic review was therefore:

1. To provide an overview of adults’ shared experiences of living with a rare condition, and explore the psychosocial consequences of this experience.
2. To address the overarching question: What experiences do people with rare diseases share?

## Materials and Methods

### Inclusion and exclusion criteria

**Figure 1:**
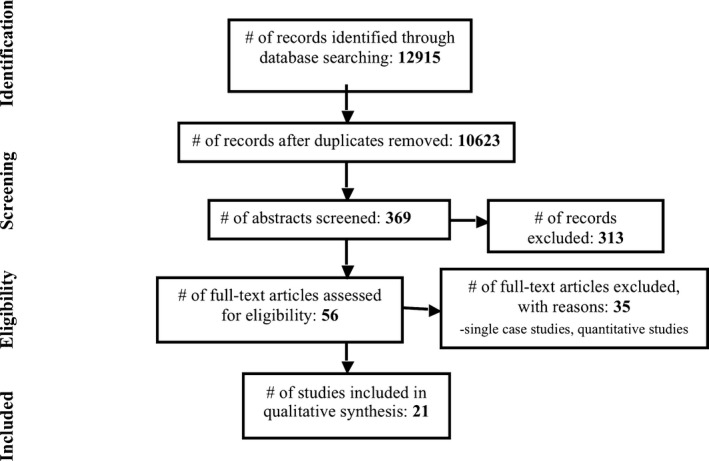
Flowchart of identified and selected articles.

A systematic review of the qualitative literature was performed, following the PRISMA statement (Moher et al. 2015). A flow chart of identified and selected articles can be found in Figure 1. All original, peer‐reviewed articles published in English, addressing adults’ experiences of living with a rare condition, based on a qualitative methodology, and published from January 2000 until December 2016 were included. Papers including children and adults or multiple groups of informants were only included if results were presented separately for the adult patient group.

Case studies and unpublished dissertations were excluded. Rare diseases involving intellectual disability were excluded. Given the enormous range of different rare conditions, studies on single diagnoses were excluded, except for four chosen diagnoses, from four different condition categories. The four diagnoses were: hemophilia (congenital bleeding disorder), phenylketonuria (congenital metabolic disorder), Fabry disease (congenital lysosomal storage disorder), and epidermolysis bullosa (congenital skin disorder). The four diagnoses were chosen based on the authors’ areas of expertise. Studies on other conditions, which included two or more diagnoses, were included, since all studies including more than one condition could potentially address shared experiences across conditions. Studies of parent's experiences or pediatric patients were excluded.

### Search strategy

The time frame for the searching and selection process was from June 2015 to December 2016. The PROSPERO International prospective register of systematic reviews was searched to be sure a similar study was not started, and a protocol for this study was published (Prospero 2016:CRD4 2016025589).

The databases searched included: MEDLINE, CINAHL, PsychINFO, ISI – Web of Knowledge, and EMBASE. MEDLINE and CINAHL were searched for any previous systematic reviews on this topic. The references lists of all selected publications were searched for additional studies.

Search words pertained to four search categories: diagnostic terms, methodological terms, psychological terms, and publication time. The Boolean operators OR were used between search terms within one category, while the operator AND was used between the categories. Diagnostic search terms were: rare dis* ‐ rare diagnos* ‐ orphan dis* ‐ fabry dis* ‐ phenylketonuria ‐ hemophilia ‐ haemophilia ‐ epidermolysis bullosa. Methodological terms were: qualitative stud* ‐ multimethod stud* ‐ phenomenolog* ‐ interview* ‐ hermeneutic ‐ psycholog* ‐ sociolog* ‐ narrative* ‐ storytelling ‐ data analysis ‐ social science ‐ quality of life ‐ anthropolog* ‐ systematic review ‐ occupational therapy ‐ physical therapy. Psychological terms were: communication ‐ satisfaction ‐ behaviour ‐ role* ‐ group process* ‐ politics ‐ government* ‐ patient* ‐ disabled ‐ disability ‐ men ‐ women ‐ empowerment ‐ sexuality ‐ compliance ‐ attitude* ‐ illness ‐ health ‐ coping ‐ knowledge ‐ information ‐ network* ‐ stress ‐ participation ‐ vulnerability ‐ symptom distress ‐ psychosocial ‐ relation* ‐ isolation ‐ stigma ‐ rehabilitation ‐ daily li* ‐ work ‐ health services ‐ social work ‐ emergency medical services ‐ wrong site surgery ‐ medical error ‐ treatment error ‐ diagnostic error ‐ patient history taking ‐ screening ‐ failure to diagnose ‐ diagnos* delay. Publication time was 2000–2016.

### Assessment of methodological quality

Search results were merged using EndNoteX6 and duplicates were removed. Two independent reviewers examined the titles and abstracts, and selected papers for full‐text reading. Both reviewers read full text of selected papers, and papers were included in the study according to the agreed criteria. Questions used to include or exclude publications after full‐text reading are shown in Appendix. Any potential disagreements between the authors were resolved through discussion. In some cases, a correspondence with the authors of included papers were needed, in order to clarify aspects related to the inclusion and exclusion criteria that were unclear, such as the age range of the participants.

### Data extraction

Two independent reviewers collected data regarding citation/contact details, methods, design, participants, setting/context, and results/findings.

### Data synthesis

Qualitative research is specific to a particular context, time and group of participants, and caution is therefore needed when generalizing results. Having this in mind, it is however possible to extract results from different qualitative studies, and synthesize findings. Several methods for synthesizing qualitative data have been recommended (Barnett‐Page and Thomas 2009), and thematic synthesis (Thomas and Harden 2008) was employed in the present review. Results were extracted from the included studies, the text was coded, and codes were grouped into meaningful categories, so‐called descriptive themes. The final synthesis presents the overall findings in analytical themes.

## Results

In total, 21 qualitative articles were included. Six articles were based on samples with hemophilia, four focused on phenylketonuria, two on Fabry disease, one on epidermolysis bullosa, and eight studies included several medical conditions in their sample. An overview of the included articles can be found in Table 1.

**Table 1:** Overview and details of included studies

Reference	Country	Diagnosis	Sample	Age range	Methodology
Barlow et al. (2007)	UK	Hemophilia	9	28–84 years	Thematic Content Analysis
Brodin et al. (2015)	Sweden	Hemophilia	14	19–80 years	Phenomenological approach
Budych et al. (2012)	Germany	Amyotrophic lateral sclerosis, Duchenne muscular dystrophy, epidermolysis bullosa, Marfan syndrome, neurodegeneration with brain iron accumulation, Wilson's disease	73	Adult patients (age not specified)	Grounded theory
Caputo (2014)	Italy	Amyotrophic lateral sclerosis, anorectal atresia, Poland syndrome, idiopathic pulmonary hypertension	32	Adult patients (age not specified)	Narrative‐based (Emotional text analysis)
Diesen (2016)	Norway	Phenylketonuria	11	20–30 years	Grounded theory
Diesen et al. (2015)	Norway	Phenylketonuria	11	20–30 years	Thematic analysis
Dures et al. (2011)	UK	Epidermolysis Bullosa	24	21–89 years	Inductive thematic analysis
Frank et al. (2007)	New Zealand	Phenylketonuria	8	31–43 years	Grounded theory
Garrino et al. (2015)	Italy	Scleroderma, Horton's disease, mixed connective tissue disease, Addison's disease, Bechet's disease, Gaucher's disease	22	21–79 years	Phenomenological approach
Gibas et al. (2008)	Canada	Fabry disease	51 female patients	22–78	Grounded theory? (not specified)
Grut and Kvam (2013)	Norway	Rare congenital medical diagnoses (not specified)	94 (51 adult patients)	20–70 years	Thematic analysis? (not specified)
Huyard (2009)	France	Cystic fibrosis, fragile X syndrome, Wilson's disease, mastocytosis, locked‐in syndrome and a sixth syndrome (Very Rare Syndrome)	29	20–47 years	French pragmatic sociology
Jaeger et al. (2015)	Sweden	Artrogryposis multiplex congenital, dysmelia, 22q11 deletion syndrome, Klinefelter syndrome	38	17–69 years	Content analysis
Kesselheim et al. (2015)	USA	Tuberous sclerosis, Phelan‐McDermid syndrome, hemophilia, pulmonary artery stenosis	9	Adult patients (personal communication with first author. Age not specified)	Grounded theory
Limperg et al. (2016)	Netherlands	Hemophilia	12	16–30 years	Thematic analysis
Nilson et al. (2012)	Canada	Hemophilia	18	18–30 years	Constant comparative method
Palareti et al. (2015)	USA and UK	Hemophilia	19	18–70 years	Bottom‐up thematic analysis
Petersen (2006)	Australia	Cystic fibrosis, hemochromatosis, hemophilia, thalassemia	21	Adult patients (age not specified)	Thematic analysis? (not specified)
Smith et al. (2014)	Canada	Hemophilia	32	40–77 years	Thematic content analysis
Vegni et al. (2010)	Italy	Phenylketonuria	20	18–25 years	Interpretative methodology
von der Lippe et al. (2016)	Norway	Fabry disease	10	24–77 years	Inductive thematic analysis

Three main themes were identified: 1) Consequences of living with a rare disorder, 2) Social aspects of living with a rare disorder, and 3) Experiences with the health care system. All main themes included subthemes, which will be subsequently described. An overview of the themes and subthemes presented in the included studies can be found in Table 2.

**Table 2:** Living with a rare disorder: Themes and subthemes presented in the included studies

Reference	Consequences			Social aspects				Health care experiences		
	Constraints and limitations	Psychol. impact	Coping strategies	What, how, and when to tell others	Stigma	Sameness and difference	Social support	Lack of knowledge	Contact with health prof.	Expert patients
Barlow et al. (2007)	X	X		X	X			X		
Brodin et al. (2015)	X	X	X		X	X		X	X	
Budych et al. (2012)		X						X		X
Caputo (2014)		X			X	X				
Diesen (2016)		X	X					X	X	
Diesen et al. (2015)	X	X	X	X	X	X	X	X		
Dures et al. (2011)	X	X	X	X	X	X	X	X		X
Frank et al. (2007)	X	X			X	X		X		X
Garrino et al. (2015)	X	X	X			X	X	X	X	
Gibas et al. (2008)	X	X						X	X	X
Grut and Kvam (2013)		X	X			X		X	X	X
Huyard 2009;							X	X	X	
Jaeger et al. (2015)	X	X			X	X		X	X	X
Kesselheim et al. (2015)		X			X			X	X	X
Limperg et al. (2016)	X	X				X	X			X
Nilson et al. (2012)			X			X				
Palareti et al. (2015)	X	X	X		X	X				X
Petersen (2006)	X	X	X	X	X	X	X			X
Smith et al. (2014)	X	X	X						X	X
Vegni et al. (2010)	X	X		X		X			X	X
von der Lippe et al. (2016)	X	X	X	X	X	X		X		

### Consequences of living with a rare disorder

Included articles described diverse physical and psychological consequences of living with a rare disorder. This theme was further categorized into three subthemes: constraints and limitations, psychological consequences, and coping strategies.

#### Constraints and limitations

More than half of the papers (14/21) acknowledged physical limitations and other constraints that were associated with the rare condition, and further impacted on psychological, emotional, and social adjustment, in addition to overall well‐being and health. Restraints could be physical (Barlow et al. 2007; Dures et al. 2011; Smith et al. 2014; Brodin et al. 2015), dietary restrictions (Frank et al. 2007; Diesen et al. 2015), pain (Gibas et al. 2008; Dures et al. 2011; Brodin et al. 2015; Garrino et al. 2015; Palareti et al. 2015; Limperg et al. 2016) or difficulties with sleep, fatigue, tiredness, and exhaustion (Petersen 2006; Jaeger et al. 2015). Constraints and physical limitations were described as impacting on work and education (Barlow et al. 2007; Gibas et al. 2008; Dures et al. 2011; Brodin et al. 2015; Garrino et al. 2015; Limperg et al. 2016), social life (Petersen 2006; Barlow et al. 2007; Gibas et al. 2008; Vegni et al. 2010; Dures et al. 2011; Brodin et al. 2015; Palareti et al. 2015), creating social attention and visibility (Diesen et al. 2015), restraints in terms of social and physical activities (Petersen 2006; Frank et al. 2007; Brodin et al. 2015; Limperg et al. 2016; von der Lippe et al. 2016), worries about finding an accepting and understanding life partner or perceiving the medical condition as a limit to other relationships (Vegni et al. 2010; Palareti et al. 2015), anxiety around planning children (Dures et al. 2011), and the constant need to manage and tailor the consequences of the rare condition with everyday life (Vegni et al. 2010).

Psychological restraints were more specifically described as dependence and lack of freedom related to the demands of treatment (Garrino et al. 2015), uncertainty about the disease evolution (Garrino et al. 2015), lack of autonomy (Vegni et al. 2010; Garrino et al. 2015), attention problems as a consequence of chronic and/or intense pain (Garrino et al. 2015; Jaeger et al. 2015), or stress and emotional distress (Smith et al. 2014; Jaeger et al. 2015).

#### Psychological impact

Most included studies (19/21) explicitly discussed the psychological and emotional impact of a rare condition on the individual. Psychological and emotional challenges were described as related to the physical limitations and constraints of the condition (Barlow et al. 2007; Smith et al. 2014; Palareti et al. 2015), attention problems (Jaeger et al. 2015), pain (Gibas et al. 2008), dependency on others (Barlow et al. 2007; Caputo 2014; Garrino et al. 2015), dependency of treatment (Diesen 2016), or the social impact of other people's lack of understanding and knowledge about the rare condition (Vegni et al. 2010; Diesen et al. 2015).

Psychological challenges were also related to the medical aspects of the condition, more specifically patients’ lack of knowledge about the medical condition (Garrino et al. 2015), uncertainty about the future (Barlow et al. 2007; Frank et al. 2007; Dures et al. 2011), uncertainty associated with the evolution, and progression of the condition (Petersen 2006; Garrino et al. 2015), treatment‐related uncertainty (Petersen 2006; Budych et al. 2012; Grut and Kvam 2013; Kesselheim et al. 2015), turnover of healthcare professionals (Vegni et al. 2010), and uncertainty regarding health professionals’ knowledge about the rare diagnosis and hence their aptitude to treat (Petersen 2006; Dures et al. 2011; Budych et al. 2012; Grut and Kvam 2013).

Some studies specifically mentioned the development or feeling of depression or psychological distress (Barlow et al. 2007; Kesselheim et al. 2015; Palareti et al. 2015), for example, due to loneliness (Dures et al. 2011), lack of social support (Kesselheim et al. 2015), hopelessness and desperation (Caputo 2014; Kesselheim et al. 2015), emotional distress and pain (Gibas et al. 2008; Palareti et al. 2015), disempowerment (Dures et al. 2011), loss of confidence (Dures et al. 2011), guilt related to the risk of passing the condition on to children (Dures et al. 2011; von der Lippe et al. 2016), frustration (Grut and Kvam 2013; Smith et al. 2014), and anxiety triggered by uncertainty and lack of knowledge about the rare condition (Frank et al. 2007; Grut and Kvam 2013; Garrino et al. 2015; Kesselheim et al. 2015). Other aspects of emotional distress were feelings of fear, anger, blame, and loss (Palareti et al. 2015).

Positive emotions were also described. Several patients described gratefulness associated with living in a country with available treatment options (Brodin et al. 2015; Limperg et al. 2016; von der Lippe et al. 2016). Positive thoughts about the medical condition were shared, however, associated with an ambiguity related to resentment and feeling of saturation regarding the need for constant treatment and surveillance from the medical treatment teams (Diesen 2016). Patients, whose treatment had successfully been supervised by their parents, shared appreciative reflections about their parents’ efforts in following through a diet in spite of the child's protests and objections (Diesen 2016).

#### Coping strategies

Half of the included papers (11/21) mentioned coping strategies that patients had developed in order to cope with the constraints and challenges associated with the rare condition, such as live day by day (Dures et al. 2011; von der Lippe et al. 2016), struggle and fight against the disorder, and rise after crises (Brodin et al. 2015; von der Lippe et al. 2016), try as far as possible to live a normal life (Smith et al. 2014; Garrino et al. 2015; Palareti et al. 2015; von der Lippe et al. 2016), never look to the future (Dures et al. 2011), keep the diagnosis a secret in order to protect themselves from other peoples’ misconceptions and perception of difference (Dures et al. 2011; Diesen et al. 2015; von der Lippe et al. 2016), act nicely and educate people with a lack of knowledge about the diagnosis, and overlook unhelpful comments (Diesen et al. 2015), develop better self‐management skills regarding the disease and its treatment (Brodin et al. 2015; Palareti et al. 2015), and chose suitable education and work (Brodin et al. 2015). People with medical conditions who experienced positive treatment effects, defined themselves as lucky and grateful, and described how this had helped them put their life outcome in perspective (Diesen 2016). Lack of trust because of experienced medical malpractice led to a need to be in control (Grut and Kvam 2013). Patients adapted to their surroundings and became an authority themselves (Dures et al. 2011). By monitoring their body and energy levels, patients learned what their limits were and when they needed to undertake treatment (Petersen 2006). Other coping strategies were that patients compared themselves with other people they perceived had more serious conditions (Petersen 2006), or involved themselves in influencing political decisions (Brodin et al. 2015).

Some coping strategies could be seen as counteractive for a positive adjustment to living with a rare disorder, such as reluctance to acknowledge having the condition (Nilson et al. 2012), avoiding to inform others about the diagnosis in situations where this would have been advisable (Brodin et al. 2015), and a wait and watch strategy for managing the consequences of the condition and its treatment (Nilson et al. 2012).

### Social aspects of living with a rare disorder

Most papers described social consequences of living with a rare diagnosis. This main theme was further categorized into four subthemes: What, how, and when to tell others; stigma; sameness and difference; and social support.

#### What, how, and when to tell others

Six of the included papers (6/21) mentioned patients’ uncertainty regarding whether to reveal the diagnosis to others or keep it a secret (Barlow et al. 2007; Dures et al. 2011; Diesen et al. 2015; von der Lippe et al. 2016), and if choosing to do so, what to tell, when and to whom (Petersen 2006; Vegni et al. 2010). Some participants described their tiredness and frustration of having to explain their diagnosis over and over again, and therefore making the choice not to reveal their conditions to new acquaintances (Diesen et al. 2015). Other patients mentioned their choice not to communicate the diagnosis to others, in order to avoid the social stigma associated with the genetic condition (von der Lippe et al. 2016).

#### Stigma

Half of the included studies (11/21) reported patients’ perceptions of stigma and social misconception, as a consequence of having a rare disorder (Petersen 2006; Barlow et al. 2007; Frank et al. 2007; Dures et al. 2011; Brodin et al. 2015; Diesen et al. 2015; Jaeger et al. 2015; Palareti et al. 2015; von der Lippe et al. 2016), and the fear of being categorized negatively (Diesen et al. 2015; von der Lippe et al. 2016), in some cases directly associated with the condition's visibility and appearance (Dures et al. 2011). In one paper, stigma‐related issues were described as being present only in semi‐close relationships, and not with close family and friends (Diesen et al. 2015). More specifically, societal attitudes and other people's lack of knowledge about the condition generated misunderstandings and misconceptions (Barlow et al. 2007; Brodin et al. 2015; Jaeger et al. 2015), feelings of discrimination, social exclusion, and isolation (Petersen 2006; Barlow et al. 2007; Frank et al. 2007; Brodin et al. 2015; Jaeger et al. 2015; Kesselheim et al. 2015; Palareti et al. 2015), or perceptions of inequality and marginalization (Caputo 2014).

#### Sameness and difference

Several papers (14/21) described themes related to sameness and difference, such as a patients’ search for normalcy (Petersen 2006; Dures et al. 2011; Nilson et al. 2012; Caputo 2014; Brodin et al. 2015; Diesen et al. 2015; Garrino et al. 2015; Jaeger et al. 2015; Limperg et al. 2016; von der Lippe et al. 2016) or the perceived experience of growing up as different from others, even within one's own family (Palareti et al. 2015). Patients needed to experience not being identified as a diagnosis, to be treated as anyone else, and described a difficult balance between sameness and difference (Vegni et al. 2010; Dures et al. 2011; Jaeger et al. 2015; Palareti et al. 2015; von der Lippe et al. 2016), and a wish for normalcy that was complicated by an impression of being on the wrong side, wrapped in cotton wool, and untouchable (Palareti et al. 2015).

Patients felt that physical constraints and limitations, such as strict dietary needs, created a perceived difference between themselves and others (Frank et al. 2007; Diesen et al. 2015), especially when the condition could not be seen by others, but still required adjustments to the participant's life and social participation (Grut and Kvam 2013; Diesen et al. 2015; Garrino et al. 2015). Some papers explicitly linked this perception of difference to the condition's social or physical visibility (Frank et al. 2007; Dures et al. 2011), whereas other papers mentioned how adherence to treatment lead to normalcy, protecting people against the confrontations with the condition's symptoms (Diesen et al. 2015; Limperg et al. 2016).

#### Social support

Less than one‐third of the included papers mentioned the importance of social support (6/21) for patients with rare conditions (Huyard 2009; Dures et al. 2011), more specifically the need to share experiences about the condition and its treatment (Petersen 2006; Dures et al. 2011; Garrino et al. 2015), and the need for emotional support (Petersen 2006; Dures et al. 2011). Support was found in family members, partners, and children (Dures et al. 2011; Diesen et al. 2015; Limperg et al. 2016), and through patient organizations and people with the same condition (Petersen 2006; Huyard 2009; Garrino et al. 2015). Support from family was described as crucial, yet associated with a complicated process in the transition period from childhood to adolescence, when gaining independence from parents and taking self‐responsibility of treatment (Limperg et al. 2016). The loss of social support from family members who understood the treatment requirements of some medical conditions was described as challenging to some young adults when reaching adulthood (Diesen et al. 2015).

### Experiences with the health care system

The third main theme was related to patients’ experiences with the health care system. This theme was further categorized into three subthemes: Lack of knowledge, contact with health professionals, and expert patients.

#### Lack of knowledge

More than half of the papers (14/21) addressed patients’ experiences of meeting health professionals with lack of knowledge about their rare diagnosis. Experiences of lack of knowledge seemed most present in local health care setting, as opposed to specialist treatment teams (Brodin et al. 2015; Diesen 2016). Lack of knowledge was described to potentially lead to a delayed diagnosis (Huyard 2009; Garrino et al. 2015; Kesselheim et al. 2015), mistreatment (Barlow et al. 2007; Dures et al. 2011), or denial of social services (Grut and Kvam 2013). Patients were critical of health professionals refusing to seek assistance to remedy the limits of their knowledge (Huyard 2009; Grut and Kvam 2013). Lack of knowledge also resulted in conflicting information about the diagnosis to the patient (Frank et al. 2007), misunderstandings (von der Lippe et al. 2016), or to inadequate and missing information (Garrino et al. 2015; Kesselheim et al. 2015). Patients felt they had the responsibility to provide information to the health professionals about the diagnosis (Gibas et al. 2008; Budych et al. 2012; Grut and Kvam 2013; Brodin et al. 2015).

The experience of health professionals’ lack of knowledge generated emotional reactions in patients, such as mistrust in doctors and/or the health care system, feelings of insecurity, and feeling of fear or anger (Barlow et al. 2007; Grut and Kvam 2013; von der Lippe et al. 2016). It also engendered a sense of desperation, isolation, and depression (Kesselheim et al. 2015), feelings of ignorance (Gibas et al. 2008), or discrimination or humiliation (Jaeger et al. 2015).

Patients also experienced lack of knowledge outside the medical ward, in work, educational, and social settings (Barlow et al. 2007; Grut and Kvam 2013; Diesen et al. 2015; von der Lippe et al. 2016), experiences that have been described in more detail in the present review's two other main categories (Consequences of living with a rare disorder and Social aspects of living with a rare disorder).

#### Contact with health professionals

Half of the papers addressed this theme (10/21). Patients described unmet needs for a holistic treatment perspective and the importance of coordinated actions between health professional (Jaeger et al. 2015). Specialized treatment centers and/or teams were associated with better patient satisfaction (Gibas et al. 2008; Brodin et al. 2015; Garrino et al. 2015). A turnover of health professionals was perceived as negative, as it created discontinuity and a need for the patient to “start all over again” (Vegni et al. 2010; Garrino et al. 2015). The need to receive understandable information about the diagnosis was highly valued by the patients (Huyard 2009; Garrino et al. 2015), in addition to being treated not as a sick body, but as a whole person (Vegni et al. 2010). Patients valued a good relationship with the health professionals involved in their treatment (Grut and Kvam 2013; Garrino et al. 2015), and expressed gratitude for being able to call experts who could provide assistance and help whenever needed (Brodin et al. 2015; P. S. Diesen 2016).

People with rare disorders experienced doctors’ and other healthcare providers’ unwillingness to get involved when the patients’ diagnosis was unknown to them (Grut and Kvam 2013). Patients were critical to health professional refusing to seek assistance to remedy the limits of their knowledge (Huyard 2009). Patients also perceived health professionals’ unwillingness toward accepting information offered by the patient, and felt that physicians were reluctant to seek out information themselves (Grut and Kvam 2013), and therefore risked to base their decisions on personal assumptions about the condition rather than knowledge (Grut and Kvam 2013). In another study (Huyard 2009), most patients were satisfied with honest and reasonable efforts from the health professionals to gradually improve their health.

Patients commented on the lack of available treatment and a lack of knowledge about whether treatment was available and possible (Kesselheim et al. 2015). Patients also described how they were willing to try out uncertain treatment options, rather than no treatment at all (Smith et al. 2014; Kesselheim et al. 2015).

#### Expert patients

Patients with rare disorders often become the expert of their own diagnosis, a theme that was addressed in half of the included papers (12/21). Patients actively sought to educate themselves by searching for information on the Internet (Frank et al. 2007; Gibas et al. 2008; Vegni et al. 2010; Budych et al. 2012; Grut and Kvam 2013; Kesselheim et al. 2015; Palareti et al. 2015; Limperg et al. 2016) and through support groups (Petersen 2006). They felt they had to be updated on their diagnosis and its treatment (Jaeger et al. 2015), and acted as advocates of their own health (Smith et al. 2014). In many instances, patients found themselves in a position of having more information about the disease than some of the health professionals on their way (Kesselheim et al. 2015). As an example, patients described possessing considerable technical knowledge, and acquiring scientific terminology about the condition (Petersen 2006), and developed a feeling of being the best ones to make decisions about their diagnosis and its treatment (Petersen 2006; Dures et al. 2011; Kesselheim et al. 2015).

## Discussion

The uniqueness of experiences and aspects of living with a rare disorder has not been reviewed systematically. Therefore, the present review systematically examined the qualitative literature pertaining to challenges associated with living with a rare disorder in adults. Findings were categorized according to three domains: consequences of living with a rare disorder, social aspects of living with a rare disorder, and experiences with the health care system and demonstrated significant shared psychological, physical, social, and emotional impacts of living with a rare medical condition across conditions.

### Psychological and physical consequences of living with a rare disorder

One of the most salient aspects of living with a rare disorder was physical and somatic constraints and limitations that were associated with the medical condition. More than a half of the included papers discussed their impact on emotional and social adjustment, in addition to on overall well‐being and health. Although some physical limitations are present across several medical conditions, other limitations are inherently related to specific conditions. Whether challenges are specific to one condition or more common, they need to be managed and tailored to everyday life, in order to restrict their psychological impact on emotional well‐being, work, education, and social life (Waldboth et al. 2016).

Further, adults with rare conditions described psychological restraints, such as a lack of autonomy and freedom due to the demands of treatment, uncertainty about the disease evolution, and emotional distress as a consequence of pain or other distressing aspects of the conditions. The social impacts of other people's lack of understanding, or misconceptions about the rare condition, were also mentioned in most articles. Psychological distress also seemed to be directly related to experiences of lack of knowledge in health care providers, which triggered patients’ uncertainty about the doctors’ aptitude to treat. The aspect of rarity of a condition in itself was not, however, described as problematic in two of the included studies (Huyard 2009; Garrino et al. 2015), as long as patients had found health professionals who were able to recognize their needs. These findings point to the importance of subjective evaluations of one's life situation, and the fact that it is the patient's feelings and beliefs regarding their condition and its treatment that determine their ability to cope with the challenges they meet, more than having a rare condition in itself (Cohen and Biesecker 2010). Interventions should therefore aim at strengthening individuals’ coping strategies (Cohen and Biesecker 2010), and address the patients’ subjective evaluation of stress, in addition to their perception of the medical follow‐up of their condition, thereby enhancing feelings of control over the consequences and impact of the disease.

Physical and psychological constraints and limitations such as pain, physical restrictions, sleep problems, or dietary restrictions, are not specific to rare conditions only. However, these challenges may become an extra burden because of the patients’ experienced lack of knowledge and understanding in society and in the health care system. Consequently, findings from the present review support the assumption that the rarity of a condition poses some unique challenges. The intrinsic challenges associated with optimizing quality of care among individuals with rare disorders, therefore suggest the need to address the lack of knowledge in health care settings and in the population in general.

### Social consequences of living with a rare disorder

Most included papers described aspects of social consequences for adults living with a rare diagnosis. Patients raised the issue of whether, when, how, and to whom they should reveal the diagnosis, and how they by doing this could risk negative social consequences, such as misconceptions, social exclusion, or stigma. Another main theme was the challenging balance between sameness and difference, and the adults’ search for an inner feeling of normalcy. Perceived difference seemed to be strongly associated with the disorder's constraints and limitations, whether these differences were visible or nonvisible to other people. Challenges seemed to be related to whether the individual felt that the consequences of the disorder labeled them as socially different, in a way that limited their social participation or required adjustments in everyday life. On the other hand, examples from the literature on rare conditions have also demonstrated that some individuals manage to find a positive balance between sameness and difference, succeeding in the task of accepting their difference as enrichment and a positive uniqueness (Beaune et al. 2004). The ambivalence of feeling both average and extraordinary has also been described in young people with more common medical conditions (Waldboth et al. 2016). Such findings emphasize the need for further research investigating intrinsic and external factors, positive as well as negative, which may be associated with the individual's emotional response to the challenges of living with a rare medical condition.

The many social aspects described by patients living with a rare condition, raises the issue of protective factors, such as social support, mentioned in several of the included articles of the present review. Importantly, social support meant the possibility to share experiences about the condition and its treatment, and receive crucial emotional support from peers and/or family. The loss of the family's social support when reaching adulthood further confirms the importance of this factor in everyday life (Diesen 2016), as also confirmed in a recent literature review on transition into adulthood when living with a medical condition (Waldboth et al. 2016).

### Lack of knowledge about rare conditions

Knowledge about rare diseases is rare, not only among the general public, but also among health care providers (Rodwell and Ayme 2015). Lack of knowledge about conditions impact on psychological and medical health may have important medical consequences. In a Swedish study about everyday impact from having a rare disease, results show that 32% of their 1660 questionnaire respondents had experienced maltreatment and 15% had experienced not being believed or acknowledged, as direct results of the lack of knowledge of the diagnosis in health care (Wallenius et al. 2009). Affected individuals can also experience problems and delays in obtaining an accurate diagnosis, and in finding reliable information (Syed et al. 2015).

Results from the present review and the literature in general show that lack of expertise among health care providers runs as a major barrier for people with rare diseases (Aymé et al. 2008; Berglund et al. 2010; Budych et al. 2012; Grut and Kvam 2013). Therefore, people with chronic conditions frequently use the Internet to locate disease information and find peer support (Ayers and Kronenfeld 2007), as was found in the present review. Several of the included studies described how patients, probably reinforced by the experienced lack of knowledge within the health care system, took responsibility for educating themselves by searching for information on the Internet. It is, however, important to be aware that the quality of the information on the Internet may be inadequate and questionable (De Martino et al. 2017; Pauer et al. 2017), and often includes little information about psychosocial adjustment and counseling (Pauer et al. 2017). On the other hand, support groups found on the Internet may provide valuable information for patients with rare diagnoses and their relatives (Pauer et al. 2017), and provide a possibility to share similar experiences and challenges (Newman et al. 2011). Peer support may therefore constitute a more supportive understanding and environment than healthcare professionals. Online community networks for people with the same rare diseases also provide emotional support, and a possibility to learn from others in a similar situation that, due to the disease incidence, would be impossible to find elsewhere (Lasker et al. 2005; Aymé et al. 2008; Gundersen 2011). Maximizing availability of information for local and specialist health professionals, in addition to patients, their families, schools, and other arenas of importance, should therefore be a priority. This should be based on existing frameworks, such as large umbrella associations that have been formed in cooperation with patient support organizations (North American National Organization for Rare Disorders (NORD) and European Organisation for Rare Diseases (EURORDIS)).

With only a handful of patients per diagnosis in each country, another challenge is that medical research progress is often slow and contains little prestige. Research activities are less frequent, and adequate treatment and medicines can be very expensive (Forman et al. 2012). An increased effort to obtain larger and representative samples could be achieved through national and international multicentre collaboration, and should be the priority of future research.

### Experiences with the health care system

Most studies illustrated the importance of patients’ experiences with the health care system. Adults described the need for a good relationship with their health care providers, to be treated with respect and as a whole person, a need for understandable information about the diagnosis, and valued health professionals who could provide assistance and help whenever needed. In contrast, patients' experienced unwillingness from the health care providers to get involved or to seek information about the rare disorder created a lack of trust. Studies have shed light on the importance of a collaborative relationship between health care providers and patients, where professionals recognize the patient as a partner in the care process (Dures et al. 2011). This medical perspective fits well with patient empowerment and patient responsibility in health care decisions, and seems even more appropriate in relation to rare conditions, given the number of studies included in the present review that described how patients became experts of their condition, in contrast to, and as a consequence of the lack of knowledge experienced in health professionals. Lack of expertise among healthcare providers therefore makes individual empowerment and emergence of the so‐called expert patient a reality and a necessity for people with rare diseases (Aymé et al. 2008; K. Budych et al. 2012). Patients who are experts on their own diagnosis have been shown to cope better (Anne Sen and Spring 2013), have better self‐rated health status, and be less dependent on hospital care (Lorig et al. 1999). Expert patient programs may be a cost‐effective solution and efficient use of scarce resources (Richardson et al. 2008), although the content of such programs has been debated (Taylor and Bury 2007). Expert patients should be seen as a value (Boulet 2016), as they fill a gap where health professionals may fall short, and they are a resource to other patients with the same diagnosis. It is, however, important that, as the patient becomes an expert, the doctor should not put more responsibility on the patient (Litzkendorf et al. 2016). Lack of knowledge about rare diseases among physicians when meeting expert patients, may be difficult for both parties. Doctors may feel challenged by lay knowledge (Prior 2003), whereas patients may test health professionals against their knowledge and expectations in order to decide whether or not to trust the physician (Mechanic and Meyer 2000). Distrust may result in less use of the healthcare system, and eventually in poorer health (Whetten et al. 2006).

Another finding related to patients’ experience with the health care system, was what seemed to be a difference between specialized treatment centers and/or teams, and local health care providers (Gibas et al. 2008; Brodin et al. 2015; Garrino et al. 2015). Centralized teams are characterized by more experience, and more knowledge about the specific conditions they are in charge of. Therefore, and not surprisingly, centralized and specialized teams may yield better patient satisfaction (Gibas et al. 2008; Brodin et al. 2015; Garrino et al. 2015; Feragen et al. 2017). This echoes professionals’ recognition of the value of interdisciplinary teamwork with patients with rare diagnoses (Dures et al. 2010). Nevertheless, previous literature has demonstrated the importance of having practitioners who communicate well and show sensitivity, personal characteristics that can be found irrespective of level of knowledge about a rare condition (Feragen et al. 2017). More research is needed, however, to evaluate difference between specialized treatment centers and local health care providers, and how responsibilities should be organized in order to secure the patient's health‐related follow‐up.

### Positive aspects of living with a rare disorder

Several papers described how adults with rare conditions had developed coping strategies that helped them cope with the everyday challenges, strategies that could strengthen the development of positive outcomes and resilience. Many coping strategies were specifically aimed at normalizing everyday life, or included the development of self‐management skills in order to deal with the social reactions to the condition. In normalizing everyday life, the patients reconstruct life and accept the situation as ordinary (Deatrick et al. 1999). Patients also described the need to build up a feeling of control in health care consultations by educating themselves about the medical aspects of their condition, as described above, and hence presenting themselves as expert patients. Others described how internal processes, such as comparing themselves with people with other more severe conditions, had helped them put their life outcome in perspective. Downward comparison is a well‐known coping strategy in terms of strengthening self‐perceptions (Taylor et al. 1990).

### Strengths and limitations

The strengths of this literature review lie in the methodological and systematic approach, investigating the life experiences of adults with rare medical conditions from a qualitative perspective. It is, however, also important to acknowledge some limitations in the present review. First, some methodological challenges were encountered. In order to restrict the large amount of quantitative and qualitative papers that were identified in the first place, the present review focused on four specific diagnoses, and aspects of living with other rare diseases that might be different from the ones included were therefore not reviewed in the present work. This limitation illustrates the need to further summarize the literature, in order to investigate similarities and differences across conditions. Future reviews could include a broader or different range of rare conditions. Literature reviews should also be conducted on the experiences of younger individuals, such as adolescents or children, in order to complete the picture provided by reviews on parents or families (Pelentsov et al. 2015; Waldboth et al. 2016). Another methodological challenge was that some studies presented quotes without the context they were a part of, complicating the synthesis of the results in the present review. Other papers presented their results as part of the discussion, also complicating the extraction of data for this review. Further, few studies explicitly explored the potential uniqueness of the rarity of a condition, investigating whether challenges that are identified have a similar or differential impact on individuals, depending of the specificity of the condition. Research needs to address whether a combination of constraints and limitations could have a differential impact or create a synergistic interaction (Vogeli et al. 2007), than predicted by the presence of the same constraints alone. Additionally, few studies explicitly tied their work to a theoretical framework. The use of theoretical framework should be a crucial component of high‐quality research and guide the development of hypotheses and methods.

## Conclusion

People with rare disorders face challenges beyond medical issues. Many of the challenges could be diminished by more knowledge and awareness about rare disorders in society, and increased focus on psychological health and coping strategies. The findings highlight the need for more research on the shared psychological and social impact of living with a rare diagnosis across diagnoses, in order to identify potential risk factors and inform clinical practice, so that the patients’ quality of life can be improved.

## Disclosure Statement

The authors declare that they have no competing interests.

## Questions to include or exclude publications after full text reading

**First author, year**:_____________________________________________

**Decision (after filling out the form):**

**Table:**

Include	⁭(All questions are answered with “YES”)
Discuss	⁭(Some questions are answered with “UNCLEAR”)
Exclude	⁭(Some questions are answered with “NO”)

**Final decision:**

**Table:**

Include	⁭(All questions are answered with “YES”)
Exclude	(Some questions are answered with “NO”) Question number:

**1: Is the study empirical and published in full text?**

**Table:**

⁭YES	NO	UNCLEAR

**2: Does the study aim to explore and/or define implications from rarity for persons living with a rare diagnosis, without being diagnose specific?**

**Table:**

⁭YES	NO	UNCLEAR

**3: Does the study aim to promote and develop knowledge about what it means to live with a rare disorder?**

**Table:**

⁭YES	⁭NO	⁭UNCLEAR

**4: Is the research design one (or more) of the following:**

**Table:**

Qualitative methods (interviews, focus groups, textual analyses)	⁭YES	⁭NO	⁭UNCLEAR
Quantitative methods (quality of life and other types of surveys)	⁭YES	⁭NO	⁭UNCLEAR
Report	⁭YES	⁭NO	⁭UNCLEAR

**5: Does the study take on a population of adolescents and/or adults with a rare disorder above the age of 12?**

**Table:**

YES	NO	⁭UNCLEAR

**6: Does the study address one or more of the following themes?**

**Table:**

Medical encounters	⁭YES	⁭NO	⁭UNCLEAR
Social service encounters	⁭YES	⁭NO	⁭UNCLEAR
Misdiagnosis, mistreatment and/or late diagnosis	⁭YES	⁭NO	⁭UNCLEAR
Retrieving information and/or use of internet in conjunction with the rare disorder	⁭YES	⁭NO	⁭UNCLEAR
Patient organisations and/or social support from peers	⁭YES	⁭NO	⁭UNCLEAR
Psychosocial implications in everyday life	⁭YES	⁭NO	⁭UNCLEAR
Insufficient level of knowledge	⁭YES	⁭NO	⁭UNCLEAR

Other, specify:_______________________________________________________________

**Comments:** __________________________________________________________________________________________________________________________________________________________________________________________________________________________________________________________________________________________________________________________________________________________________________________________________________________________________________________________________

## References

[mgg3315-bib-0001] Anne Sen, B. , and H. Spring . 2013 Mapping the information‐coping trajectory of young people with long term illness: an evidence based approach. J. Doc. 69:638–666.

[mgg3315-bib-0002] Ayers, S. L. , and J. J. Kronenfeld . 2007 Chronic illness and health‐seeking information on the Internet. Health (London) 11:327–347. 17606698 10.1177/1363459307077547

[mgg3315-bib-0003] Aymé, S. , A. Kole , and S. Groft . 2008 Empowerment of patients: lessons from the rare diseases community. Lancet 371:2048–2051. 18555918 10.1016/S0140-6736(08)60875-2

[mgg3315-bib-0004] Barlow, J. H. , J. Stapley , and D. R. Ellard . 2007 Living with haemophilia and von Willebrand's: a descriptive qualitative study. Patient Educ. Couns. 68:235–242. 17904328 10.1016/j.pec.2007.06.006

[mgg3315-bib-0005] Barnett‐Page, E. , and J. Thomas . 2009 Methods for the synthesis of qualitative research: a critical review. BMC Med. Res. Methodol. 9:59. 19671152 10.1186/1471-2288-9-59 PMC3224695

[mgg3315-bib-0006] Beaune, L. , C. R. Forrest , and T. Keith . 2004 Adolescents’ perspectives on living and growing up with Treacher Collins syndrome: a qualitative study. Cleft Palate Craniofac. J. 41:343–350. 15222792 10.1597/02-158.1

[mgg3315-bib-0007] Berglund, B. , A.‐C. Mattiasson , and I. Randers . 2010 Dignity not fully upheld when seeking health care: experiences expressed by individuals suffering from Ehlers‐Danlos syndrome. Disabil. Rehabil. 32:1–7. 19925271 10.3109/09638280903178407

[mgg3315-bib-0008] Boulet, L. P. 2016 The expert patient and chronic respiratory diseases. Can. Respir. J. 2016:9454506. 27445572 10.1155/2016/9454506 PMC4904534

[mgg3315-bib-0009] Boycott, K. M. , M. R. Vanstone , D. E. Bulman , and A. E. MacKenzie . 2013 Rare‐disease genetics in the era of next‐generation sequencing: discovery to translation. Nat. Rev. Genet. 14:681–691. 23999272 10.1038/nrg3555

[mgg3315-bib-0010] Brodin, E. , K. S. Sunnerhagen , F. Baghaei , and M. Tornbom . 2015 Persons with Haemophilia in Sweden‐ experiences and strategies in everyday life. A single centre study. PLoS ONE 10:e0139690. 26431432 10.1371/journal.pone.0139690 PMC4592191

[mgg3315-bib-0011] Budych, K. , T. M. Helms , and C. Schultz . 2012 How do patients with rare diseases experience the medical encounter? Exploring role behavior and its impact on patient‐physician interaction. Health Policy 105:154–164. 22464590 10.1016/j.healthpol.2012.02.018

[mgg3315-bib-0012] Caputo, A. 2014 Exploring quality of life in Italian patients with rare disease: a computer‐aided content analysis of illness stories. Psychol. Health Med. 19:211–221. 23651424 10.1080/13548506.2013.793372

[mgg3315-bib-0013] Cohen, J. S. , and B. B. Biesecker . 2010 Quality of life in rare genetic conditions: a systematic review of the literature. Am. J. Med. Genet. A, 152a:1136–1156. 20425818 10.1002/ajmg.a.33380 PMC3113481

[mgg3315-bib-0014] De Martino, I. , R. D'Apolito , A. S. McLawhorn , K. A. Fehring , P. K. Sculco , and G. Gasparini . 2017 Social media for patients: benefits and drawbacks. Curr. Rev. Musculoskelet. Med. 10:141–145. https://doi.org/10.1007/s12178-017-9394-7. 28110391 10.1007/s12178-017-9394-7 PMC5344865

[mgg3315-bib-0015] Deatrick, J. A. , K. A. Knafl , and C. Murphy‐Moore . 1999 Clarifying the concept of normalization. Image J. Nurs. Sch. 31:209–214. 10528448 10.1111/j.1547-5069.1999.tb00482.x

[mgg3315-bib-0016] Diesen, P. S. 2016 “I feel lucky” ‐ gratitude among young adults with phenylketonuria (PKU). J. Genet. Couns. 25:1002–1009. 26888542 10.1007/s10897-015-9931-8

[mgg3315-bib-0017] Diesen, P. S. , I. Wiig , L. Grut , and B. F. Kase . 2015 Betwixt and between being healthy and ill: the stigma experienced by young adults with phenylketonuria. Scand. J. Disabil. Res. 17:321–334.

[mgg3315-bib-0018] Dodge, J. A. , T. Chigladze , J. Donadieu , Z. Grossman , F. Ramos , A. Serlicorni , et al. 2011 The importance of rare diseases: from the gene to society. Arch. Dis. Child. 96:791–792. 20705719 10.1136/adc.2010.193664

[mgg3315-bib-0019] Dures, E. , M. Morris , K. Gleeson , and N. Rumsey . 2010 ‘You're whatever the patient needs at the time’: the impact on health and social care professionals of supporting people with epidermolysis bullosa. Chron. Ill. 6:215–227. 10.1177/1742395310377006 20663801

[mgg3315-bib-0020] Dures, E. , M. Morris , K. Gleeson , and N. Rumsey . 2011 The psychosocial impact of epidermolysis bullosa. Qual. Health Res. 21:771–782. doi:10.1177/1049732311400431. 21343430 10.1177/1049732311400431

[mgg3315-bib-0021] Feragen, K. B. , N. Rumsey , A. Heliovaara , B. M. Boysen , E. C. Johannessen , C. Havstam , et al. 2017 Scandcleft randomised trials of primary surgery for unilateral cleft lip and Palate: 9. Parental report of social and emotional experiences related to their 5‐year‐old child's cleft diagnosis. J. Plast. Surg. Hand Surg. 51:73–80. 28218553 10.1080/2000656X.2016.1254643

[mgg3315-bib-0022] Forman, J. , D. Taruscio , V. A. Llera , L. A. Barrera , T. R. Coté , C. Edfjäll , et al. 2012 The need for worldwide policy and action plans for rare diseases. Acta Paediatr. 101:805–807. 22519914 10.1111/j.1651-2227.2012.02705.x PMC3443385

[mgg3315-bib-0023] Frank, N. , R. Fitzgerald , and M. Legge . 2007 Phenylketonuria‐the lived experience. N. Z. Med. J. 120:U2728. 17891216

[mgg3315-bib-0024] Garrino, L. , E. Picco , I. Finiguerra , D. Rossi , P. Simone , and D. Roccatello . 2015 Living with and treating rare diseases: experiences of patients and professional health care providers. Qual. Health Res. 25:636–651. 25667160 10.1177/1049732315570116

[mgg3315-bib-0025] Gibas, A. L. , R. Klatt , J. Johnson , J. T. Clarke , and J. Katz . 2008 Disease rarity, carrier status, and gender: a triple disadvantage for women with Fabry disease. J. Genet. Couns. 17:528–537. 18931895 10.1007/s10897-008-9179-7

[mgg3315-bib-0026] Grut, L. , and M. H. Kvam . 2013 Facing ignorance: people with rare disorders and their experiences with public health and welfare services. Scand. J. Disabil. Res. 15:20–32.

[mgg3315-bib-0027] Gundersen, T. 2011 ‘One wants to know what a chromosome is’: the internet as a coping resource when adjusting to life parenting a child with a rare genetic disorder. Sociol. Health Illn. 33:81–95. 20937053 10.1111/j.1467-9566.2010.01277.x

[mgg3315-bib-0028] Haffner, M. E. , J. Whitley , and M. Moses . 2002 Two decades of orphan product development. Nat. Rev. Drug Discov. 1:821–825. 12360259 10.1038/nrd919

[mgg3315-bib-0029] Huyard, C. 2009 What, if anything, is specific about having a rare disorder? Patients’ judgements on being ill and being rare. Health Expect. 12:361–370. 19840131 10.1111/j.1369-7625.2009.00552.x PMC5060508

[mgg3315-bib-0030] Institute of Medicine (US) Committee on Accelerating Rare Diseases Research and Orphan Product Development . 2010 Field M. J., Boat T. F., eds. Rare Diseases and Orphan Products: Accelerating Research and Development. National Academies Press (US), Washington (DC) Available from: https://www.ncbi.nlm.nih.gov/books/NBK56189/ doi: https://doi.org/10.17226/12953 21796826

[mgg3315-bib-0031] Jaeger, G. , A. Rojvik , and B. Berglund . 2015 Participation in society for people with a rare diagnosis. Disabil. Health J. 8:44–50. 25164983 10.1016/j.dhjo.2014.07.004

[mgg3315-bib-0032] Kesselheim, A. S. , S. McGraw , L. Thompson , K. O'Keefe , and J. J. Gagne . 2015 Development and use of new therapeutics for rare diseases: views from patients, caregivers, and advocates. Patient 8:75–84. 25362528 10.1007/s40271-014-0096-6

[mgg3315-bib-0033] Kole, A. , and F. Faurisson . 2009 The voice of 12,000 patients: experiences and expectations of rare disease patients on diagnosis and care in Europe. Available at http://www.eurordis.org/IMG/pdf/voice_12000_patients/EURORDISCARE_FULLBOOKr.pdf [Stand: 07.04. 2009].

[mgg3315-bib-0034] Lasker, J. N. , E. D. Sogolow , and R. R. Sharim . 2005 The role of an online community for people with a rare disease: content analysis of messages posted on a primary biliary cirrhosis mailinglist. J. Med. Internet Res. 7:e10. 15829472 10.2196/jmir.7.1.e10 PMC1550634

[mgg3315-bib-0035] Limperg, P. , M. Peters , E. Gibbons , M. Coppens , C. Valk , M. Grootenhuis , et al. 2016 Themes in daily life of adolescents and young adults with congenital bleeding disorders: a qualitative study. Haemophilia 22:e330–e333. 27217221 10.1111/hae.12961

[mgg3315-bib-0037] von der Lippe, C. , J. C. Frich , A. Harris , and K. N. Solbraekke . 2016 Experiences of being heterozygous for fabry disease: a qualitative study. J. Genet. Couns. 25:1085–1092. 26948256 10.1007/s10897-016-9941-1

[mgg3315-bib-0038] Litzkendorf, S. , A. Babac , D. Rosenfeldt , F. Schauer , T. Hartz , V. Lührs , et al. 2016 Information needs of people with rare diseases‐what information do patients and their relatives require? J. Rare Disord. Diagn. Ther. 2:40.

[mgg3315-bib-0039] Lorig, K. R. , D. S. Sobel , A. L. Stewart , B. W. Jr Brown , A. Bandura , P. Ritter , et al. 1999 Evidence suggesting that a chronic disease self‐management program can improve health status while reducing hospitalization: a randomized trial. Med. Care 37:5–14. 10413387 10.1097/00005650-199901000-00003

[mgg3315-bib-0040] Mechanic, D. , and S. Meyer . 2000 Concepts of trust among patients with serious illness. Soc. Sci. Med. 51:657–668. 10975226 10.1016/s0277-9536(00)00014-9

[mgg3315-bib-0041] Moher, D. , L. Shamseer , M. Clarke , D. Ghersi , A. Liberati , M. Petticrew , et al. 2015 Preferred reporting items for systematic review and meta‐analysis protocols (PRISMA‐P) 2015 statement. Syst. Rev. 4:1. 25554246 10.1186/2046-4053-4-1 PMC4320440

[mgg3315-bib-0042] Molster, C. , D. Urwin , L. Di Pietro , M. Fookes , D. Petrie , S. van der Laan , et al. 2016 Survey of healthcare experiences of Australian adults living with rare diseases. Orphanet J. Rare Dis. 11:30. 27012247 10.1186/s13023-016-0409-z PMC4806449

[mgg3315-bib-0043] Nelson, P. A. 2009 Qualitative approaches in craniofacial research. Cleft Palate Craniofac. J. 46:245–251. 19642761 10.1597/08-121.1

[mgg3315-bib-0044] Newman, M. W. , D. Lauterbach , S. A. Munson , P. Resnick , and M. E. Morris . 2011 It's not that I don't have problems, I'm just not putting them on Facebook: challenges and opportunities in using online social networks for health. Paper presented at the Proceedings of the ACM 2011 conference on Computer supported cooperative work.

[mgg3315-bib-0045] Nilson, J. , C. Schachter , K. Mulder , M. Hahn , M. Steele , P. Hilliard , et al. 2012 A qualitative study identifying the knowledge, attitudes and behaviours of young men with mild haemophilia. Haemophilia 18:e120–e125. 22171673 10.1111/j.1365-2516.2011.02714.x

[mgg3315-bib-0046] Nutt, S. , and L. Limb . 2011 Survey of patients’ and families’ experiences of rare diseases reinforces calls for a rare disease strategy. Soc. Care Neurodis. 2:195–199.

[mgg3315-bib-0047] Palareti, L. , S. Poti , F. Cassis , F. Emiliani , D. Matino , and A. Iorio . 2015 Shared topics on the experience of people with haemophilia living in the UK and the USA and the influence of individual and contextual variables: Results from the HERO qualitative study. Int. J. Qual. Stud. Health Well‐being 10:28915. 26578360 10.3402/qhw.v10.28915 PMC4649019

[mgg3315-bib-0048] Pauer, F. , S. Litzkendorf , J. Gobel , H. Storf , J. Zeidler , and J. M. Graf von der Schulenburg . 2017 Rare diseases on the internet: an assessment of the quality of online information. J. Med. Internet Res. 19:e23. 28100442 10.2196/jmir.7056 PMC5288561

[mgg3315-bib-0049] Pelentsov, L. J. , T. A. Laws , and A. J. Esterman . 2015 The supportive care needs of parents caring for a child with a rare disease: a scoping review. Disabil. Health J. 8:475–491. 25959710 10.1016/j.dhjo.2015.03.009

[mgg3315-bib-0050] Petersen, A. 2006 The best experts: the narratives of those who have a genetic condition. Soc. Sci. Med. 63:32–42. 16431006 10.1016/j.socscimed.2005.11.068

[mgg3315-bib-0051] Prior, L. 2003 Belief, knowledge and expertise: the emergence of the lay expert in medical sociology. Sociol. Health Illn. 25:41–57. 14498929 10.1111/1467-9566.00339

[mgg3315-bib-0052] Richardson, G. , A. Kennedy , D. Reeves , P. Bower , V. Lee , E. Middleton , et al. 2008 Cost effectiveness of the Expert Patients Programme (EPP) for patients with chronic conditions. J. Epidemiol. Community Health 62:361–367. 18339831 10.1136/jech.2006.057430

[mgg3315-bib-0053] Rodwell, C. , and S. Ayme . 2015 Rare disease policies to improve care for patients in Europe. Biochem. Biophys. Acta., 1852:2329–2335. 25725454 10.1016/j.bbadis.2015.02.008

[mgg3315-bib-0054] Smith, N. , C. Bartholomew , and S. Jackson . 2014 Issues in the ageing individual with haemophilia and other inherited bleeding disorders: understanding and responding to the patients’ perspective. Haemophilia 20:e1–e6. 24118548 10.1111/hae.12278

[mgg3315-bib-0055] Strauss, A. , and J. Corbin . 1990 Basics of qualitative research. Vol. 15 Sage, Newbury Park, CA.

[mgg3315-bib-0056] Syed, A. M. , R. Camp , C. Mischorr‐Boch , F. Houyez , and A. R. Aro . 2015 Policy recommendations for rare disease centres of expertise. Eval. Program Plann. 52:78–84. 25935363 10.1016/j.evalprogplan.2015.03.006

[mgg3315-bib-0057] Taylor, D. , and M. Bury . 2007 Chronic illness, expert patients and care transition. Sociol. Health Illn. 29:27–45. 17286704 10.1111/j.1467-9566.2007.00516.x

[mgg3315-bib-0058] Taylor, S. E. , B. P. Buunk , and L. G. Aspinwall . 1990 Social comparison, stress, and coping. Pers. Soc. Psychol. Bull. 16:74–89.

[mgg3315-bib-0059] Thomas, J. , and A. Harden . 2008 Methods for the thematic synthesis of qualitative research in systematic reviews. BMC Med. Res. Methodol. 8:45. 18616818 10.1186/1471-2288-8-45 PMC2478656

[mgg3315-bib-0060] Vegni, E. , L. Fiori , E. Riva , M. Giovannini , and E. A. Moja . 2010 How individuals with phenylketonuria experience their illness: an age‐related qualitative study. Child Care Health Dev. 36:539–548. 19735270 10.1111/j.1365-2214.2009.01000.x

[mgg3315-bib-0061] Vogeli, C. , A. E. Shields , T. A. Lee , T. B. Gibson , W. D. Marder , K. B. Weiss , et al. 2007 Multiple chronic conditions: prevalence, health consequences, and implications for quality, care management, and costs. J. Gen. Intern. Med. 22(Suppl 3):391–395. 18026807 10.1007/s11606-007-0322-1 PMC2150598

[mgg3315-bib-0062] Waldboth, V. , C. Patch , R. Mahrer‐Imhof , and A. Metcalfe . 2016 Living a normal life in an extraordinary way: a systematic review investigating experiences of families of young people's transition into adulthood when affected by a genetic and chronic childhood condition. Int. J. Nurs. Stud. 62:44–59. 27450665 10.1016/j.ijnurstu.2016.07.007

[mgg3315-bib-0063] Wallenius, E. , K. Möller , and B. Berglund . 2009 Everyday impact of having a rare diagnosis: a questionnaire study. Vård i Norden 3:13–17.

[mgg3315-bib-0064] Wastfelt, M. , B. Fadeel , and J. I. Henter . 2006 A journey of hope: lessons learned from studies on rare diseases and orphan drugs. J. Intern. Med. 260:1–10. 16789973 10.1111/j.1365-2796.2006.01666.x

[mgg3315-bib-0065] Whetten, K. , J. Leserman , R. Whetten , J. Ostermann , N. Thielman , M. Swartz , et al. 2006 Exploring lack of trust in care providers and the government as a barrier to health service use. Am. J. Public Health 96:716–721. 16507725 10.2105/AJPH.2005.063255 PMC1470533

